# Variability in Adaptive Resistance of *Salmonella* Typhimurium to Sublethal Levels of Antibiotics

**DOI:** 10.3390/antibiotics11121725

**Published:** 2022-12-01

**Authors:** Jirapat Dawan, Juhee Ahn

**Affiliations:** 1 Department of Biomedical Science, Kangwon National University, Chuncheon 24341, Gangwon, Republic of Korea; 2 Institute of Bioscience and Biotechnology, Kangwon National University, Chuncheon 24341, Gangwon, Republic of Korea

**Keywords:** adaptive response, antibiotic susceptibility, fitness, cross-resistance, *Salmonella*

## Abstract

This study was designed to evaluate the adaptive resistance of *Salmonella* Typhimurium under continuous sublethal selective pressure. *Salmonella* Typhimurium ATCC 19585 (ST^ATCC^) and *S*. Typhimurium CCARM 8009 (ST^CCARM^) were sequentially cultured for 3 days at 37 °C in trypticase soy broth containing 1/2 × MICs of cefotaxime (CEF^1/2^), chloramphenicol (CHL^1/2^), gentamicin (GEN^1/2^), and polymyxin B (POL^1/2^). The ST^ATCC^ and ST^CCARM^ exposed to CEF^1/2^, CHL^1/2^, GEN^1/2^, and POL^1/2^ were evaluated using antibiotic susceptibility, cross-resistance, and relative fitness. The susceptibilities of ST^ATCC^ exposed to GEN^1/2^ and POL^1/2^ were increased by a 2-fold (gentamicin) and 8-fold (polymyxin B) increase in minimum inhibitory concentration (MIC) values, respectively. The MIC values of ST^CCARM^ exposed to CEF^1/2^, CHL^1/2^, GEN^1/2^, and POL^1/2^ were increased by 4-fold (cefotaxime), 2-fold (chloramphenicol), 2-fold (gentamicin), and 8-fold (polymyxin B). The highest heterogeneous fractions were observed for the ST^ATCC^ exposed to CEF^1/2^ (38%) and POL^1/2^ (82%). The ST^CCARM^ exposed to GEN^1/2^ was cross-resistant to cefotaxime (*p* < 0.05), chloramphenicol (*p* < 0.01), and polymyxin B (*p* < 0.05). The highest relative fitness levels were 0.92 and 0.96, respectively, in ST^ATCC^ exposed to CEF^1/2^ and ST^CCARM^ exposed to POL^1/2^. This study provides new insight into the fate of persistent cells and also guidance for antibiotic use.

## 1. Introduction

Over the last few decades, the emergence and spread of antibiotic resistance have become some of the top threats to global health [1,2]. Bacteria can acquire resistance mechanisms under antibiotic selective pressures [3]. In addition, bacterial populations consist of persisters as tolerant subpopulations [3]. Indeed, persistence, tolerance, and resistance are characterized by the bacterial growth rates during the antibiotic exposure. Bacterial persistence is a metabolically inactive state including dormant cells under an antibiotic stress condition. Tolerance is a slow-growing state, and resistance is an actively growing state in the presence of antibiotic stress [4]. Antibiotic treatment failure is mainly due to antibiotic-resistance-conferring mutations, antibiotic resistance acquisition via horizontal gene transfer (HGT), and the formation of persistent cells [5]. Antibiotic resistance in bacteria is genetically acquired, but persistence is characterized as phenotypic heterogeneity [6]. The mechanisms of antibiotic resistance in bacteria include enzymatic inactivation of antibiotics, alteration in binding affinity of antibiotics, modification of membrane permeability, and use of alternative metabolic pathways [7]. Efflux pumps, DNA damage, stress responses, molecular degradation, and toxin–antitoxin (TA) molecules can affect persister cell formation [8]. However, the formation of persister cells is not clearly understood. Bacteria persistence is a reversible and transient phenomenon that can survive under stressful conditions [9].

Antibiotic tolerance in persister cells is due to dormancy, leading to fewer active target sites for antibiotics [10]. Persister cells are divided into two types; one is type I persistence in stationary phase (triggered persistence), and the other is type II persistence in exponential phase (spontaneous persistence) [10]. Spontaneous persistence depends on a stochastic switch to persister cells which rarely occurs, while triggered persistence relies on metabolic and environmental stresses [11,12]. Persister cells can revitalize in favorable conditions [10]. Hence, not only antibiotic resistance but also tolerance and persistence can cause serious public health problems [1]. However, persister cells are relatively less often investigated and are underestimated with regard to the risk of infection. Furthermore, it is still unknown whether tolerance and persistence as bacterial survival strategies can affect the development of resistance [3]. Therefore, this study aimed to characterize in terms of antibiotic susceptibility, cross-resistance, and relative fitness the *Salmonella* Typhimurium wild-type strain (ST^ATCC^), which serves as the original parent strain, and the antibiotic-resistant strain (ST^CCARM^), which encodes pre-resistome genes that can respond to several stress conditions [13], by exposing them to sublethal levels of antibiotics.

## 2. Results and Discussion

In the early stage of antibiotic discovery, antibiotics became a successful treatment for bacterial infections. However, the overuse and misuse of antibiotics have become major causes of the development of antibiotic resistance in bacteria [14]. Bacteria evolve survival strategies under unfavorable antibiotic treatments [9]. For instance, persistence is an intrinsic defense system which protects bacteria from stress-induced damage [15]. Bacterial persisters are subpopulations that reversibly tolerate antibiotics and cause recalcitrant chronic infections [11,12,16,17,18,19,20]. However, bacterial persistence is overlooked due to a lack of information [2]. Therefore, differentiation between persister cells and antibiotic-resistant cells is needed for the design of effective antibiotic therapy [12].

### 2.1. Antibiotic Susceptibility and Heterogeneous Subpopulation under Continuous Sublethal Selective Pressure

**Figure 1:**
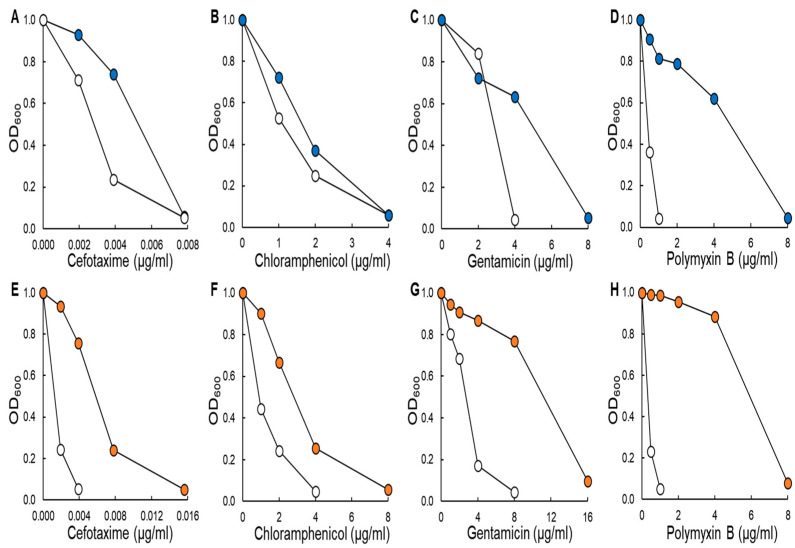
Antibiotic susceptibilities of *Salmonella* Typhimurium ATCC 19585 (ST^ATCC^); (**A**–**D**) and *S*. Typhimurium CCARM 8009 (ST^CCARM^: **E**–**H**) exposed to a half minimum inhibitory concentration (MIC) of cefotaxime (CEF^1/2^; **A**,**E**), chloramphenicol (CHL^1/2^; **B**,**F**), gentamicin (GEN^1/2^; **C**,**G**), and polymyxin B (POL^1/2^; **E**,**H**) for 0 (control; ○, ○) and 3 (●, ●) days. The optical density (OD) of ST^ATCC^ and the ST^CCARM^ untreated control and after exposure to CEF^1/2^, CHL^1/2^, GEN^1/2^, and POL^1/2^ for 3 days was measured after incubating at 37 °C for 18 h.

**Figure 2:**
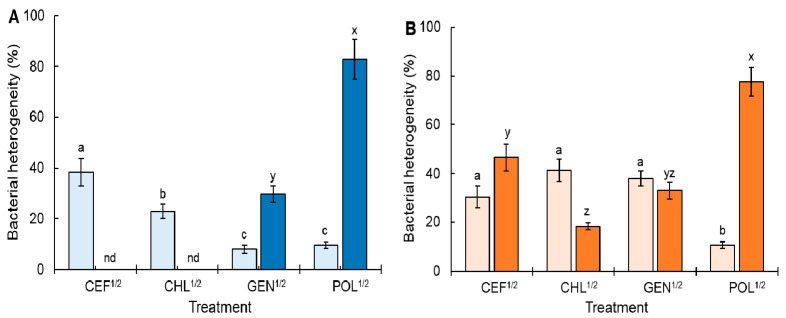
Frequencies of persistence (■, ■) and resistance (■, ■) in *Salmonella* Typhimurium ATCC 19585 (ST^ATCC^; **A**) and *S*. Typhimurium CCARM 8009 (ST^CCARM^: **B**) exposed to a half minimum inhibitory concentration (MIC) of cefotaxime (CEF^1/2^), chloramphenicol (CHL^1/2^), gentamicin (GEN^1/2^), and polymyxin B (POL^1/2^). Different letters (a–c) on the bars show significant differences among treatments within persistence at *p* < 0.05, and different letters (x–z) on the bars show significant difference among treatments within resistance at *p* < 0.05. nd represents not determined.

Antibiotics including cefotaxime, chloramphenicol, gentamicin, and polymyxin B were used to evaluate the antibiotic susceptibilities of ST^ATCC^ and ST^CCARM^ exposed to a half MICs of antibiotics for 3 days at 37 °C (CEF^1/2^, CHL^1/2^, GEN^1/2^, and POL^1/2^, respectively) (Figure 1). For ST^ATCC^, no changes in the MICs of cefotaxime and chloramphenicol were observed after exposure to CEF^1/2^ and CHL^1/2^ (Figure 1A,B), corresponding to the absence of antibiotic resistance (Figure 2A). ST^ATCC^ is antibiotic-sensitive bacteria with the absence of pre-resistance genes [21]. Thus, ST^ATCC^ may not transform from the antibiotic-sensitive to the antibiotic-resistant phenotype. In addition, the most common mechanisms of resistance to β-lactam and chloramphenicol in bacteria are the production of antibiotic-degrading enzymes such as β-lactamases and the presence of efflux pumps [21]. On the other hand, other studies also mentioned that β-lactams may act as persistence effectors through the induction of SOS responses [22]. Thus, this observation suggests that cefotaxime and chloramphenicol are more likely to be associated with the persistence formation in ST^ATCC^ rather than resistance development. However, the susceptibilities of ST^ATCC^ to gentamicin and polymyxin B were decreased after exposure to GEN^1/2^ and POL^1/2^, showing a 2-fold and 8-fold increase in MIC values, respectively (Figure 1C,D). For ST^CCARM^, all treatments, CEF^1/2^, CHL^1/2^, GEN^1/2^, and POL^1/2^, showed increased MIC values of cefotaxime (4-fold; Figure 1E), chloramphenicol (2-fold; Figure 1F), gentamicin (2-fold; Figure 1G), and polymyxin B (8-fold; Figure 1H). Bacteria are easily adapted to subinhibitory concentrations of antibiotics, resulting in enhanced antibiotic resistance [6]. In general, enhanced persistence level is attributed to serial antibiotic exposure, which can also lead to the development of bacterial tolerance to different classes of antibiotics [23].

The bacterial heterogeneity of ST^ATCC^ and ST^CCARM^ exposed to CEF^1/2^, CHL^1/2^, GEN^1/2^, and POL^1/2^ was evaluated with the formation of persister and resistant cells (Figure 2). Compared to the control, the highest persistence fraction was observed for ST^ATCC^ exposed to CEF^1/2^ (38%), followed by CHL^1/2^ (23%). This is in good agreement with a previous report which stated that the continuous exposure of bacteria to antibiotics can promote the formation of persister cells [24]. The formation of persisters is induced by the inhibition of replication, transcription, and translation [8,16,25] and is also influenced by experimental conditions and antibiotic classes; thereby, it is known as a complex phenomenon [26,27]. For instance, antibiotics such as tetracycline, aminoglycosides, β-lactams, and fluoroquinolones act as persistence effectors that inhibit DNA replication and protein synthesis [28,29]. In addition, β-lactams are involved in the induction of an SOS response through lexA/recA regulators [22]. These regulators play an important role in the expression of the class I toxin–antitoxin (TA) system, resulting in bacterial growth inhibition and persister cell formation [30]. Furthermore, the SOS response is also associated with the formation of small colony variants (SCVs) which typically enhance intracellular residence in host cells, promote persistence, and increase the risk of antibiotic tolerance and resistance [23]. Persister cells are characterized by morphological variation (small colony and cell wall-deficient state), metabolic alterations (reduced ATP production and cell membrane modification), gene expression changes (fitness- and survival-associated regulators and TA mechanism), and stress responses (pH and oxygen) [29,31]. ST^ATCC^ exposed to POL^1/2^ showed the highest resistance percentage (83%), followed by GEN^1/2^ (30%) (Figure 2A). Resistant cells were not detected in ST^ATCC^ exposed to CEF^1/2^ and CHL^1/2^. The ST^CCARM^ cells exposed to CEF^1/2^, CHL^1/2^, and GEN^1/2^ were more than 30% persister cells (Figure 2B). Similar to ST^ATCC^, the highest resistance fraction was observed in ST^CCARM^ exposed to POL^1/2^ (78%).

*Salmonella* Typhimurium has several resistance mechanisms to antimicrobial peptides, such as polymyxin B, which regulate the activation of the two-component regulatory PmrAB system and the increased secretion of outer membrane vesicles containing antimicrobial agents from the bacterial envelope [32]. This implies that the ST^ATCC^ and ST^CCARM^ strains used in this study are able to activate the resistance mechanisms that can lead to easy adaptation to polymyxin B and a large proportion of polymyxin-B-resistant cells. This observation suggests that the PmrAB operon and outer membrane vesicles are more likely to be associated with resistance development than persistence formation [32]. Persistence can be an intermediator for the evolution of antibiotic resistance [1]. Therefore, persister cells can involve the emergence of antibiotic-resistant development [6]. The survival curves of persister cells exposed to antibiotics showed characteristic biphasic behaviors, representing a rapid decline in a majority of the vulnerable populations at the early phase, followed by a slow decrease in a minority of the tolerant populations at the late phase [17,33].

### 2.2. Cross-Resistance under Continuous Sublethal Selective Pressure

**Figure 3:**
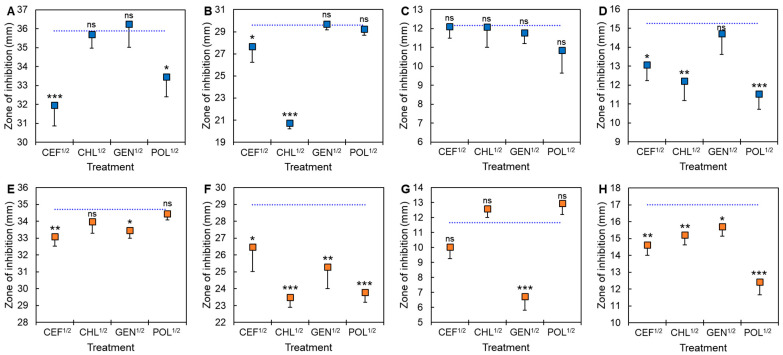
Cross-resistance of *Salmonella* Typhimurium ATCC 19585 (ST^ATCC^; **A**–**D**) and *S*. Typhimurium CCARM 8009 (ST^CCARM^: **E**–**H**) exposed to a half minimum inhibitory concentration (MIC) of cefotaxime (CEF^1/2^), chloramphenicol (CHL^1/2^), gentamicin (GEN^1/2^), and polymyxin B (POL^1/2^) to cefotaxime (**A**,**E**), chloramphenicol (**B**,**F**), gentamicin (**C**,**G**), and polymyxin B (**D**,**H**). The dotted lines represent untreated ST^ATCC^ and ST^CCARM^. ns indicates no significant difference and *, **, and *** denote significance differences at *p* < 0.05, *p* < 0.01, and *p* < 0.001, respectively.

The development of cross-resistance by ST^ATCC^ and ST^CCARM^ exposed to CEF^1/2^, CHL^1/2^, GEN^1/2^, and POL^1/2^ was evaluated using different classes of antibiotic disks (Figure 3). All treatments, including CEF^1/2^, CHL^1/2^, GEN^1/2^, and POL^1/2^, showed significant resistance to the same antibiotics, cefotaxime, chloramphenicol, gentamicin, and polymyxin B, respectively, except for ST^ATCC^ exposed to GEN^1/2^ against gentamicin (Figure 3C). ST^ATCC^ cells exposed to POL^1/2^ (*p* < 0.05) were cross-resistant to cefotaxime (Figure 3A), CEF^1/2^ (*p* < 0.05) to chloramphenicol (Figure 3B), and CEF^1/2^ (*p* < 0.05), GEN^1/2^ (*p* < 0.001), and POL^1/2^ (*p* < 0.001) to polymyxin B (Figure 3D). The cross-resistance of ST^CCARM^ exposed to GEN^1/2^ was observed with cefotaxime (Figure 3E), chloramphenicol (Figure 3F), gentamicin (Figure 3G), and polymyxin B (Figure 3H). The enhanced cross-resistance of ST^ATCC^ exposed to CEF^1/2^, CHL^1/2^, and POL^1/2^ and ST^CCARM^ exposed to CEF^1/2^, CHL^1/2^, GEN^1/2^, and POL^1/2^ with cefotaxime, chloramphenicol, gentamicin, and polymyxin B resulted from the continuous sublethal selective pressure [34,35]. In general, persisters are responsible for de novo mutation when exposed to antibiotics [36]. Moreover, cell-wall-deficient spheroplasts formed under β-lactam antibiotic treatment are responsible for enhanced antibiotic tolerance and persistence [17,37]. The antibiotic tolerance of persister cells is also attributed to enhanced efflux pump activity [38]. It demonstrates that metabolically active cells can induce persister formation through the efflux pump [18]. This implies that persisters highly express multidrug efflux pumps which decrease intracellular antibiotic concentrations, resulting in enhanced antibiotic resistance [38,39]. Although enhanced susceptibility was not noticeable in this study, ST^ATCC^ exposed to GEN^1/2^ (Figure 3A) and ST^CCARM^ exposed to CHL^1/2^ and POL^1/2^ (Figure 3G) showed increased susceptibility to cefotaxime and gentamicin, respectively. This result is known as negative cross-resistance, where the acquisition of resistance to one antibiotic results in increased susceptibility to another antibiotic [40]. Persister cells become susceptible to antibiotics due to the awakening of persisters triggered by environmental signals [8,41,42]. Therefore, selection inversion approaches can enhance the antibiotic susceptibility of antibiotic-resistant bacteria [43].

### 2.3. Relative Fitness under Continuous Sublethal Selective Pressure

**Figure 4:**
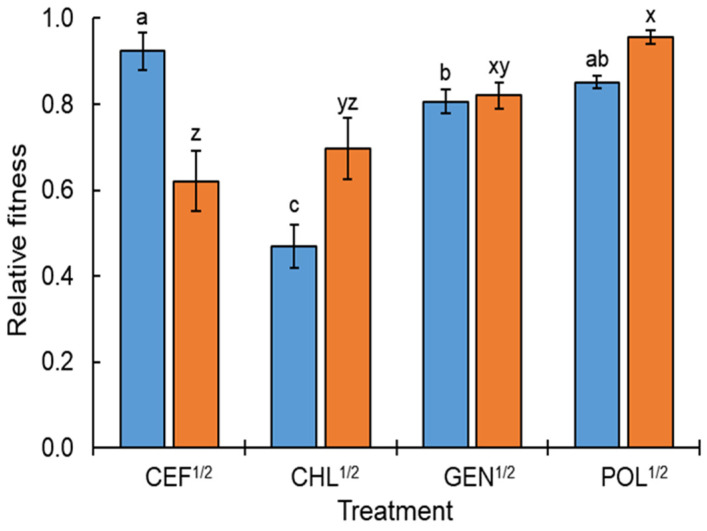
Relative fitness of *Salmonella* Typhimurium ATCC 19585 (ST^ATCC^; ■) and *S*. Typhimurium CCARM 8009 (ST^CCARM^; ■) exposed to a half minimum inhibitory concentration (MIC) of cefotaxime (CEF^1/2^), chloramphenicol (CHL^1/2^), gentamicin (GEN^1/2^), and polymyxin B (POL^1/2^). Different letters (a–c) on the bars show significant differences among treatments within persistence at *p* < 0.05, and different letters (x–z) on the bars show significant differences among treatments within resistance at *p* < 0.05.

The relative fitness was determined to evaluate the cost of resistance of ST^ATCC^ and ST^CCARM^ exposed to CEF^1/2^, CHL^1/2^, GEN^1/2^, and POL^1/2^ (Figure 4). The ST^ATCC^ cells exposed to CEF^1/2^ showed the highest relative fitness level (0.92), while those exposed to CHL^1/2^ showed the lowest relative fitness level (0.47), indicating the highest fitness cost. The highest fitness level achieved was that of ST^CCARM^ exposed to POL^1/2^ (0.96), followed by that achieved by GEN^1/2^ (0.82) and CHL^1/2^ (0.70) (Figure 4). Antibiotic-susceptible bacterial cells outcompete antibiotic-resistant cells that have low fitness when cultured in the absence of antibiotics [21]. Low fitness costs are likely to acquire antibiotic resistance in bacteria [44]. Therefore, a low fitness cost might be associated with the increase in the frequency of antibiotic resistance and cross-resistance of ST^ATCC^ and ST^CCARM^ exposed to CEF^1/2^, CHL^1/2^, GEN^1/2^, and POL^1/2^ when compared to other classes of antibiotics (Figure 3). As a result, this allows bacteria to survive under antibiotic exposure, implying that bacteria can quickly adapt to new environments by optimizing the fitness [42]. Fitness is an important factor for maintaining antibiotic resistance [21]. The growth of antibiotic-resistant bacteria depends on the fitness cost in the absence of antibiotics [3]. The fitness of persister cells is reduced in favorable growth conditions, but their phenotypic heterogeneity allows them to adapt to stressful conditions [45]. The resistance acquired at sublethal concentrations of antibiotics shows higher fitness than that acquired under lethal concentrations [46]. Compensatory mutations can help antibiotic-resistant bacteria to survive in the absence of antibiotics [44]. In contrast, antibiotic-tolerant persisters revert to the antibiotic-susceptible wild type when exposed to antibiotic-free media [33]. The antibiotic susceptibility of persister cells can be increased when treated with metabolic enhancers such as daptomycin with glucose [47], fluoroquinolones with L-serine [48], and gentamicin with L-arginine [49], resulting in elevated uptake of proton motive force-dependent antibiotics [50].

## 3. Materials and Methods

### 3.1. Bacterial Strains and Culture Conditions

Strains of *Salmonella enterica* subsp. *enterica* serovar Typhimurium ATCC 19585 (ST^ATCC^) and *S*. Typhimurium CCARM 8009 (ST^CCARM^) were obtained from the American Type Culture Collection (ATCC, Manassas, VA, USA) and the Culture Collection of Antibiotic Resistant Microbes (CCARM, Seoul, Korea), respectively. ST^ATCC^ and ST^CCARM^ were used as representative of wild-type, antibiotic-sensitive and antibiotic-resistant strains, respectively [13]. All strains were cultured in trypticase soy broth (TSB; BD; Becton, Dickinson and Co., Sparks, MD) at 37 °C for 20 h and washed three times with phosphate-buffered saline (PBS; pH 7.2) by a centrifugation at 6000× *g* for 15 min at 4 °C. The collected cells were diluted with PBS to 10^8^ CFU/mL for further assays.

### 3.2. Antibiotic Susceptibility Assay

The antibiotic susceptibilities of ST^ATCC^ and ST^CCARM^ were determined according to the microbroth dilution assay [51]. The antibiotics used in this study are listed in Table 1. Antibiotic stock solutions were prepared by dissolving in ethanol for chloramphenicol and distilled water for cefotaxime, gentamicin, and polymyxin B to obtain a final concentration of 10.24 mg/mL. Each antibiotic stock solution was serially (1:2) diluted with fresh TSB in 96-well microtiter plates (BD Falcon, San Jose, CA, USA). Approximately 10^5^ CFU/mL of ST^ATCC^ or ST^CCARM^ was inoculated in the microtiter plates. After inoculation, the plates were incubated at 37 °C for 18 h to measure optical density (OD) at 600 nm using a microplate reader (BioTek Instruments, Inc., Winooski, VT, USA) and to determine minimum inhibitory concentration (MIC) where no visible growth was observed.

### 3.3. Analysis of Heterogeneous Cell Populations

ST^ATCC^ and ST^CCARM^ were successively cultured in TSB containing 1/2 × MICs of cefotaxime (CEF^1/2^; 0.004 and 0.002 μg/mL), chloramphenicol (CHL^1/2^; 2 and 2 μg/mL), gentamicin (GEN^1/2^; 2 and 4 μg/mL), and polymyxin B (POL^1/2^; 0.5 and 0.5 μg/mL) for 3 days at 37 °C. The antibiotic-treated ST^ATCC^ and ST^CCARM^ cells (CEF^1/2^, CHL^1/2^, GEN^1/2^, and POL^1/2^; 10 μL each) were daily resuspended in 1 mL of TSB containing a half MIC of each antibiotic. After exposure to antibiotics, the growths of untreated control and ST^ATCC^ and ST^CCARM^ exposed to CEF^1/2^, CHL^1/2^, GEN^1/2^, and POL^1/2^ as a function of antibiotic concentration were used to estimate the fractions of heterogeneous cells. The area under curve was calculated using sub-MIC and resistance-selective windows [52]. The selective windows of ST^ATCC^ and ST^CCARM^ exposed to CEF^1/2^, CHL^1/2^, GEN^1/2^, and POL^1/2^ were compared with those of the untreated control to measure heterogeneity.

### 3.4. Disk Diffusion Susceptibility Test

The cross-resistance of ST^ATCC^ and ST^CCARM^ exposed to CEF^1/2^, CHL^1/2^, GEN^1/2^, and POL^1/2^ was evaluated using antibiotic disk diffusion assay. Antibiotic disks (Oxoid Ltd., Hampshire, United Kingdom) used in this study were cefotaxime (30 μg), chloramphenicol (30 μg), gentamicin (10 μg), and polymyxin B (300 μg). The antibiotic-exposed ST^ATCC^ and ST^CCARM^ cells were spread on Mueller–Hinton agar plates. After the plates were dried for 10 min, antibiotic disks were placed on the surface of agar plates, and the plates were incubated at 37 °C for 24 h. The diameter of the inhibition zone was measured using a digital vernier caliper (The L.S. Starrett Co., Athol, MA, USA).

### 3.5. Relative Fitness

The relative fitness of ST^ATCC^ and ST^CCARM^ exposed to CEF^1/2^, CHL^1/2^, GEN^1/2^, and POL^1/2^ was determined by culturing in antibiotic-free TSB at 37 °C for 24 h. The relative fitness was calculated to evaluate the cost of resistance of ST^ATCC^ and ST^CCARM^ exposed to CEF^1/2^, CHL^1/2^, GEN^1/2^, and POL^1/2^, which were expressed as the ratio of the growth of antibiotic-treated cells to antibiotic-untreated control cells cultured in antibiotic-free TSB.

### 3.6. Statistical Analysis

Data were analyzed using the Statistical Analysis System (SAS) software. All analyses were performed with three biological replicates. The general linear model (GLM) and Fisher’s least significant difference (LSD) procedures were used to determine mean differences at 5%, 1%, and 0.1% levels of significance.

## 4. Conclusions

In conclusion, the most significant finding was that bacterial persistence can cause a significant impact on the development of antibiotic resistance. Bacteria exposed to antibiotic selection pressure promote the emergence and spread of antibiotic resistance. Antibiotic resistance and persistence are characterized by transferable genetic variation and transient phenotypic variations, respectively, in bacteria. Although bacterial persistence can contribute to the evolution of antibiotic resistance, antibiotic-tolerant bacteria show growth defects in the absence of antibiotics. The population-based study of persistence is not sufficient to specifically identify and characterize persister cells in mixed populations. Thus, single-cell-based approaches need to accurately identify specific markers and distinguish persister cells. The evolution of antibiotic persistence cannot go unnoticed in clinical practice. It is essential to understand the mechanisms of persister formation when designing effective antibiotic strategies.

## Figures and Tables

**Table 1:** Antimicrobial property of antibiotics used in this study.

Class	Antibiotic	Spectrum	Activity	Affinity	Inhibitory Mechanisms
Cephems	Cefotaxime	Broad	Cidal	Hydrophilic	Cell wall synthesis
Aminoglycosides	Gentamicin	Narrow	Cidal	Hydrophilic	Protein synthesis (30S)
Glycopeptides	Polymyxin B	Narrow	Cidal	Hydrophilic	Membrane permeability
Phenicols	Chloramphenicol	Broad	Static	Hydrophobic	Protein synthesis (50S)
